# Operando Impedance Spectroscopy Informed Dynamic Internal Resistance Compensation Mitigates Bubble-Induced Distortions of the Applied Potential

**DOI:** 10.1021/acselectrochem.5c00368

**Published:** 2025-10-14

**Authors:** Blaž Tomc, Miha Hotko, Aleš Marsel, Nik Maselj, Maja Svete, Luka Suhadolnik, Marjan Bele, Pedro Farinazzo Bergamo Dias Martins, Dušan Strmčnik, Miran Gaberšček, Nejc Hodnik

**Affiliations:** † Laboratory for Electrocatalysis, Department of Materials Chemistry, National Institute of Chemistry, Ljubljana 1000, Slovenia; ‡ University of Nova Gorica, Nova Gorica 5000, Slovenia; § University of Ljubljana, Faculty of Chemistry and Chemical Technology, Ljubljana 1000, Slovenia; ∥ Institute of Metals and Technology, Ljubljana 1000, Slovenia

**Keywords:** electrochemical impedance spectroscopy, operando, IR compensation, bubbles, stability, electrochemical CO_2_ reduction

## Abstract

Bubble formation during gas-evolving electrochemical reactions disrupts potential control and obscures intrinsic catalyst–performance relationships. While conventional strategies to mitigate this issue have relied on surface engineering or cell design, here we introduce a dynamic internal resistance (IR) compensation approach that adapts to bubble-induced fluctuations in real time. This was achieved by employing (i) operando electrochemical impedance spectroscopy (EIS), which provided the electrolyte resistance (*R*_Ohm_) parameter continuously, and (ii) a Python control loop to extract EIS data and dynamically adjust the IR compensation. This self-correcting approach effectively suppressed bubble-induced artifacts during prolonged electrochemical CO_2_ reduction (ECO_2_R) on copper, enabling accurate performance evaluation and reliable assessment of catalyst instability under realistic operating conditions. We argue that such control is essential in ECO_2_R stability studies, where even minor potential shifts can obscure intrinsic catalyst–performance relationships and hinder mechanistic insight into degradation.

## Graphical abstract

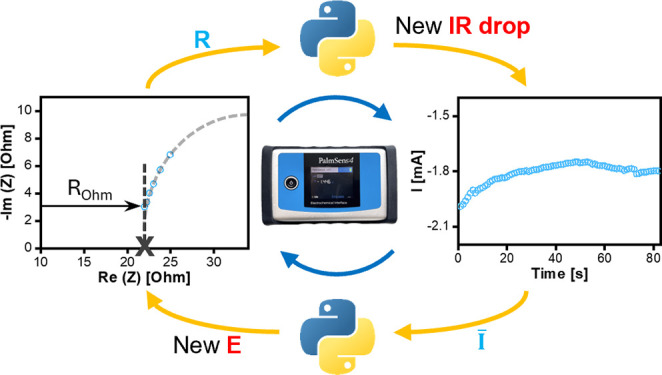

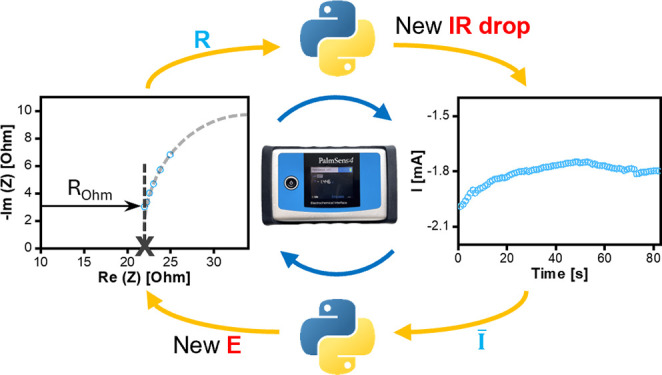

## Introduction

Electrochemical systems are pivotal to energy conversion and storage technologies,^1^ yet their performance assessment is often complicated by measurement artifacts. In gas-evolving reactions such as electrochemical CO_2_ reduction (ECO_2_R) or hydrogen evolution reaction (HER), bubble formation at the electrode surface disrupts ion transport and introduces alterations in the electrolyte resistance (*R*_Ohm_).^2^ These random changes obscure the actual potential applied to the catalyst–electrolyte interface, leading to energy losses,^3–5^ signal fluctuations,^6–14^ and distortions of reliable structure–performance relationships.^2,15^

Efforts to mitigate bubble effects have primarily focused on surface modification, electrode design, or cell engineering.^5–8,16–18^ While such strategies can improve bubble detachment and reduce blockage, they cannot eliminate the intrinsic stochastic nature of bubble formation. Recent studies have shown that bubble dynamics predominantly alter *R*_Ohm_, whereas charge-transfer kinetics remain largely unaffected.^2^ This highlights the importance of applying precise internal resistance (IR) compensation (*I* × *R*_Ohm_) to ensure that the intended potential is delivered to the electrode interface. However, in prolonged studies, static IR compensation is insufficient: as bubbles form and detach unpredictably over time, *R*_Ohm_ continuously fluctuates,^19^ leading to under- or over-compensation. This is especially problematic in reactions such as ECO_2_R, where even a few tens of mV difference on the applied potential may result in a substantial difference in product selectivity,^15^ emphasizing the need for adaptive approaches.

Electrochemical impedance spectroscopy (EIS) offers a powerful operando tool for capturing *R*_Ohm_ directly^19–28^ under reaction conditions^29^ in a non-invasive manner.^30^ By applying a small-amplitude potential or a current perturbation across a range of frequencies, EIS enables the deconvolution of complex electrochemical behavior into basic processes with distinct relaxation times (Scheme 1).^30–32^ The two most commonly observed parameters in EIS studies are ohmic *R*_Ohm_ and charge transfer resistances (*R*_CT_; Scheme 1c).^32^ *R*_Ohm_ reflects ion transport limitations (e.g., electrolyte resistance), while *R*_CT_ captures the kinetics of electrochemical reactions (Scheme 1d).^29^

**Scheme 1:**
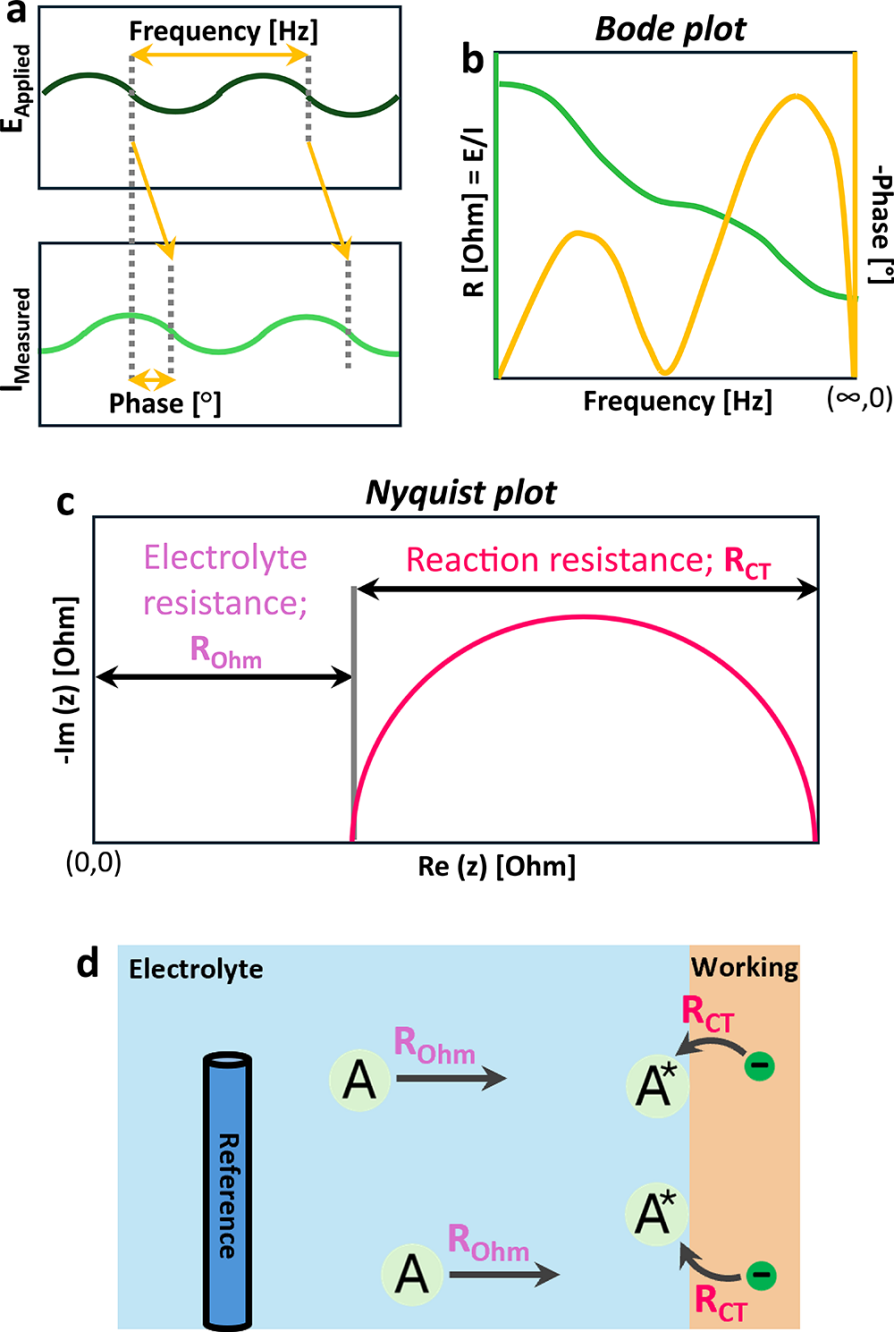
Conceptual Illustration of an Idealized EIS Response^a^

a a) A sinusoidal potential perturbation yields a delayed current response with a characteristic phase shift. b) The system’s response across different frequencies forms a Bode plot, showing amplitude and phase behavior. c) Transformation into a Nyquist plot reveals semicircle(s) associated with *R*_CT_, while *R*_Ohm_ appears as the high-frequency intercept on the real axis. d) Schematic depiction of the physical origin of *R*_Ohm_ and *R*_CT_.

However, despite its advantages, EIS has rarely been used to actively correct electrochemical measurements in real time. Conventional use is limited to post-experiment analysis or diagnostic studies, leaving potential control vulnerable to bubble-induced distortions during operation. To achieve accurate and artifact-free assessment of catalyst performance, it is essential to integrate the continuous information obtained from EIS directly into the experimental control.

Here, we demonstrate a self-correcting approach that couples operando EIS with a Python-based feedback loop to dynamically adapt the IR compensation to bubble-induced *R*_Ohm_ fluctuations. In this strategy, the potentiostat settings are updated in real-time, ensuring that the intended potential is consistently applied at the catalyst–electrolyte interface, even under highly dynamic conditions caused by bubble formation. The method’s capabilities were demonstrated on copper-catalyzed ECO_2_R, where, by eliminating bubble-induced artifacts, our approach enables reliable evaluation of catalyst activity and selectivity evolutions under realistic operating conditions. More broadly, this framework provides a generalizable route for improving measurement accuracy in various other gas-evolving electrochemical systems.

## Experimental Section

Copper foil (Jima Copper, 99.8%) was used as received, without additional surface preparation. For each experiment, a new 1.5 × 1.5 cm copper foil sheet was used. It was mounted in a gas-tight, self-made, “Sandwich type” 3-electrode electrochemical cell made of Teflon (Figure S1).^33,34^ The reference electrode was Leak-Free Ag/AgCl (Alvatek), and platinum foil was used for the anode. The Selemion membrane separated the cathodic and anodic compartments, which were both filled with 1.2 mL of 0.1 M potassium bicarbonate (KHCO_3_, 99.7% Honeywell, USA). CO_2_ (99,998% Messer, Austria) was bubbled in a cathodic compartment with a constant flow of 2.8 g/h.

Before each measurement, the electrolyte was pre-bubbled with CO_2_ for 30 min in a separate beaker. The reference electrode was measured in this electrolyte vs. the reversible hydrogen electrode (RHE, HydroFlex, Gaskatel) until the potential stabilized for ±1 mV. After saturation and potential stability, the reference electrode was inserted into the cell. The potential was applied a few seconds before the electrolyte was added to the cell. After the experiment, the reference electrode potential was measured vs. RHE again. Measurements were accepted if the reference electrode drift was within ±5 mV before and after the experiment (Table S1). Electrochemical measurements were conducted by utilizing a PalmSens4 potentiostat. The applied potential was calculated as E_CA_ = E_desired_ + E_Ag/AgCl_, and a 100% IR compensation was applied.

Gas products were analyzed online every 10 min using an SRI 8610C gas chromatograph (GC) equipped with flame ionization and thermal conductivity detectors. The calculated Faradaic efficiencies (FE) were normalized to 100% to account for bubble-induced fluctuations, as shown in Figure S2. Liquid products were not analyzed in the present study.

The detailed explanation of scripting using PSTrace to employ operando EIS and the presentation of the Python code are presented in Section S2.

## Results and Discussion

Preliminary chronoamperometry (CA) and chronopotentiometry (CP) experiments under static IR compensation immediately revealed the challenges of potential control during gas-evolving electrolysis (Figure 1a,b). A 2-hour CA at −1.0 V vs RHE with 25 Ohm IR compensation showed geometric current density (*j*_Geo_) and selectivity fluctuations with strong drifts detected. Similarly, in a CP experiment at −4.5 mA, oscillations in the applied potential and selectivity were observed, though with different drift behavior. In both cases, the trends were inconsistent and difficult to reproduce (Figure S9), suggesting that bubble dynamics strongly distorted the applied potential. Direct visualization in a half-cell configuration confirmed that bubbles formed randomly and dynamically on the copper surface during operation (Figure 1c). The *j*_Geo_ and potential jumps, highlighted with brown dotted arrows in Figure 1a,b, directly coincided with bubble detachment. Schematically illustrated in Figure 1d, bubbles nucleate on the catalyst surface and obstruct ion transport, reaching maximum interference just before detachment. Once detached, the obstruction is relieved, restoring ion migration. This cyclic process is reflected in the electrochemical profiles: (i) *j*_Geo_ decreases until a sudden jump to a higher value, and (ii) the applied overpotential gradually increases until the bubble detaches, after which a lower overpotential is sufficient to sustain the same current density.

**Figure 1:**
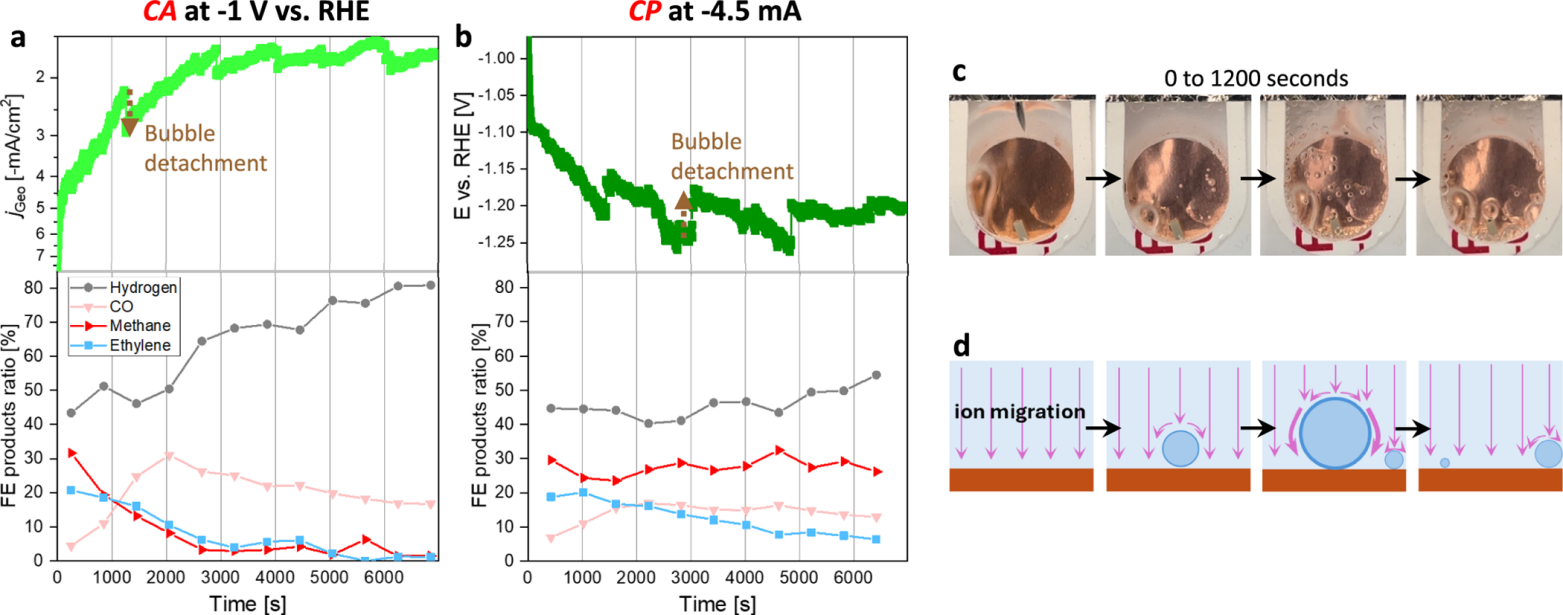
a) Normal CA at −1 V vs. RHE and a constant IR compensation of 25 Ohm with the measured *j*_Geo_ and ECO_2_R selectivity. b) Normal CP at −4.5 mA applied current with the measured applied potential and ECO_2_R selectivity. c) The half-cell setup shows bubble formation during operation. d) Illustration of bubble formation and its impact on ion migration.

Beyond the observed fluctuations of up to 50 mV and more than 25% variation in *j*_Geo_, we also detected pronounced drifts of the values. During CA, *j*_Geo_ shifted toward lower absolute values, whereas in CP, evolution manifested as a drift toward higher overpotentials. One possible explanation is a drift in *R*_Ohm_, for example, arising from the presence of immobile bubbles that obstruct ion transport. In parallel, the ECO_2_R selectivity trends further indicated ongoing changes within the system. To correctly resolve the origin of these performance shifts, it is essential that the potential at the catalyst–electrolyte interface remains constant. In principle, CA with appropriate IR compensation should provide such control; however, as bubbles continuously evolve, no static compensation approach can adequately ensure these conditions.

To further probe the role of bubbles in driving fluctuations and ECO_2_R performance evolution, we performed operando EIS measurements using a custom PSTrace script. The experimental sequence consisted of repeated 2 min loops with (i) CA at −1.025 V vs. RHE under 18 Ohm IR-compensated (bubble-free path; Figure S10) CA, followed by (ii) an EIS measurement at the same potential. This enabled non-destructive tracking of *R*_Ohm_ throughout the experiment and confirmed, as anticipated, that this resistance varied dynamically over time (Figure 2a). While fluctuations were evident, no growth evolution in *R*_Ohm_ was detected that could explain the progressive decline in *j*_Geo_. Consequently, the CP approach proved completely unreliable under these conditions (Figure 1b), as selectivity shifts stemmed predominantly from changes in the applied interfacial potential. By contrast, the CA experiment with 25 Ohm static IR compensation (Figure 1a) provided a reasonable approximation when compared to the average *R*_Ohm_ measured in Figure 2a, therefore, yielding a qualitatively reliable picture of ECO_2_R selectivity evolution. Notably, the first two selectivity points in CA and CP experiments produced nearly identical product ratios. This agreement arose because CP at −4.5 mA initially applied an overpotential equivalent to around −1.0 V vs. RHE (−1.1 V + 4.5 mA* ∼ 25 Ohm), matching the CA conditions when *j*_Geo_ was close to -4.5 mA (Figure 1a). However, toward the end of the experiment, CP at −4.5 mA imposed substantially higher overpotentials compared to the −1.0 V vs. RHE CA, leading to increasingly distorted selectivity trends.

**Figure 2:**
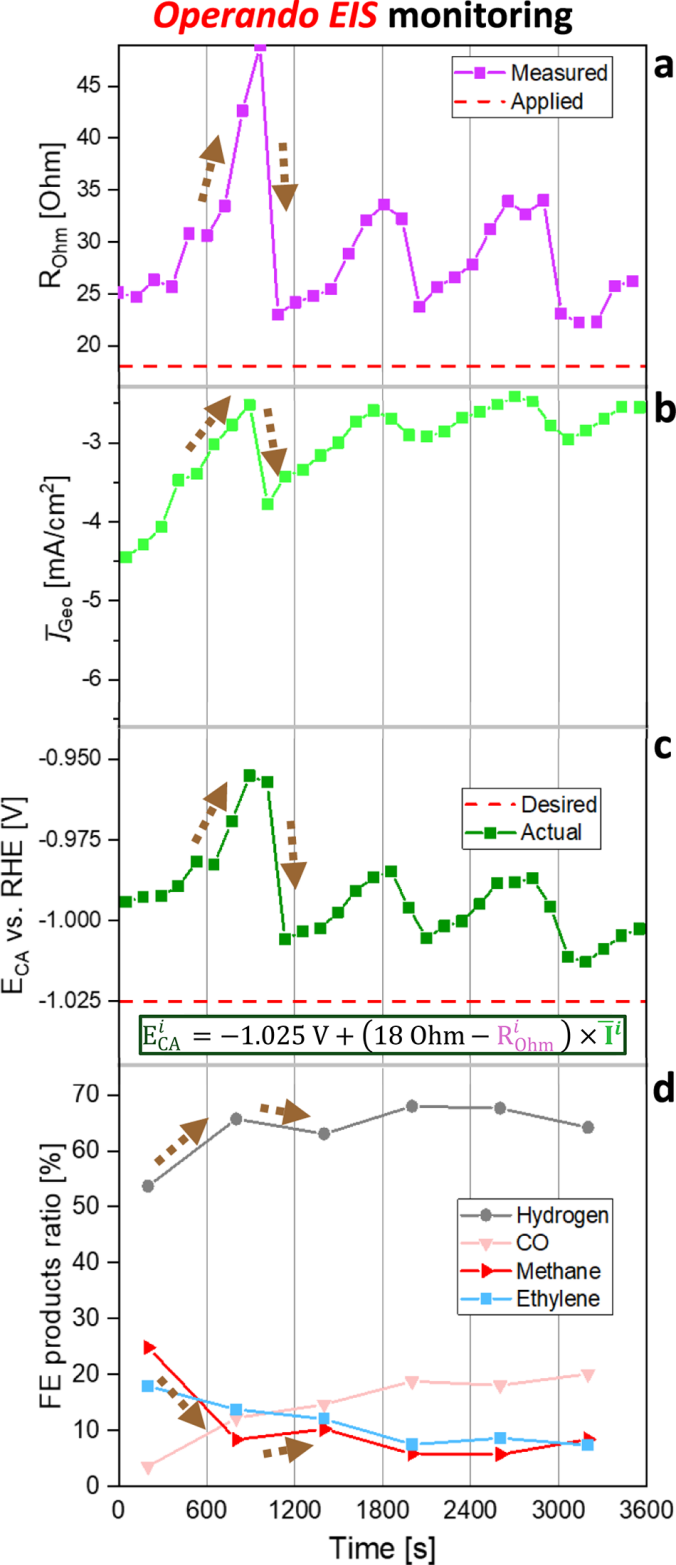
a–d) Operando EIS monitored ECO_2_R stability at −1.025 V vs. RHE and static 18 Ohm IR compensation. The applied potential (c) was calculated via the equation presented in the Figure. The brown dotted arrows represent how a major bubble formation and its detachment affect the *R*_Ohm_, *j̅*_Geo_, actual applied potential at the interface, and ECO_2_R product selectivity.

Furthermore, the evolution of *R*_Ohm_ directly explained the fluctuating nature of the average geometric current density (*j̅*_Geo_) observed in Figure 2b. As *R*_Ohm_ increased and subsequently decreased, *j̅*_Geo_ followed the same trend, as highlighted by the brown dashed arrows (Figure 2a,b). Post-mortem 100% IR compensation of the applied potential (Figure 2c) confirmed that the interfacial potential fluctuated in the same manner. Correspondingly, HER and methane selectivities mirrored these fluctuations (Figure 2d). While this further supports the intricate interplay between bubbles and the electrochemical reactions (Figure 1d), the resulting ECO_2_R selectivity evolution was unreliable. Moreover, *R*_Ohm_ never reached the bubble-free path (Figure S10), confirming that some bubbles were always present on the surface. Thus, the applied conditions in Figure 2 not only varied substantially throughout the experiment due to bubble dynamics, but they were also consistently misapplied throughout the experiment. An additional test, where the static IR compensation was increased to 30 Ohm in an attempt to counteract *R*_Ohm_ changes, revealed that the stochastic nature of bubble dynamics instead caused periods of substantial IR overcompensation (Figure S11). At such times, the potentiostat imposed extreme potential oscillations, ultimately worsening control of the interfacial potential and the selectivity evolution.

These findings establish dynamic IR compensation as a necessary advancement in electrochemistry to achieve reliable control of the applied potential at the catalyst–electrolyte interface. Since *R*_Ohm_ can be obtained in real time via operando EIS, and the correct IR compensation must follow its evolution,^2^ we developed a Python script to monitor the operando EIS results, extract *R*_Ohm_ values, and continuously adjust the IR compensation of the CA in real time (Figure 3a). The operando EIS loop was modified to include two additional 2s CA steps, providing sufficient time for real-time parameter updates. To ensure a consistent applied potential, both the EIS and short CA steps were corrected using the latest *R*_Ohm_ and the average current (I̅), as depicted on Figure 3a. Furthermore, care was taken during changes of the techniques to keep an uninterrupted applied potential. Full details of the script are provided in Section S2.

**Figure 3:**
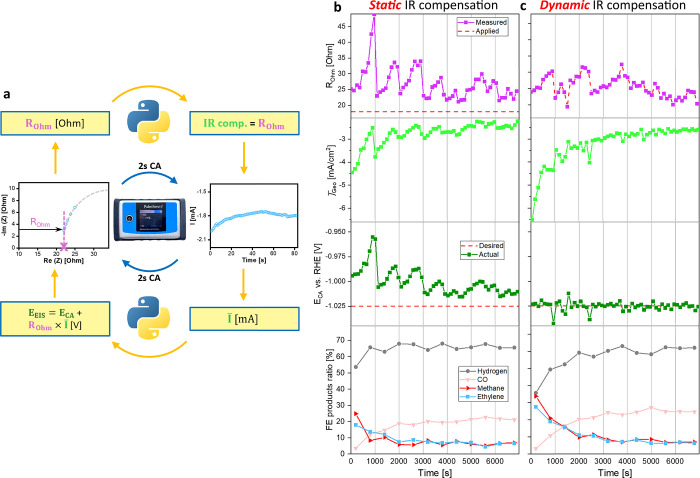
a) Workflow diagram of the program used in c): the potentiostat cycles through techniques while the Python script adjusts parameters in real time. b) Operando EIS monitored ECO_2_R stability at −1.025 V vs. RHE and static 18 Ohm IR compensation. c) ECO_2_R stability test at −1.025 V vs. RHE and dynamic IR compensation enforced via the approach presented in (c).

By adapting in real time to bubble-induced *R*_Ohm_ fluctuations, the self-correcting operando EIS consistently maintained an applied potential of −1.025 V vs. RHE (Figure 3c). Although minor fluctuations persisted because the IR compensation was updated every 2 min, the control of the potential was markedly superior to the static IR compensation case. As a result, the evolving ECO_2_R activity and selectivity were reliably measured at the true intended potential throughout the entire experiment. In contrast, static IR compensation led to systematic underapplication of potential, reflected in the lower *j̅*_Geo_ (−4.5 vs. −6.5 mA at the start of the measurement) and altered ECO_2_R selectivities, including a ∼20% decrease in HER FE ratio and ∼10% increases in both methane and ethylene FE ratios at the first measured point. Such deviations could easily be mistaken for intrinsic changes in catalytic activity or selectivity, when in reality they arise solely from potential misapplication.

These discrepancies highlight dynamic compensation as a necessary tool to eliminate bubble-induced artifacts and ensure that the applied potential remains consistent throughout prolonged electrolysis. Without this feedback control, even small stochastic shifts in *R*_Ohm_ accumulate into significant errors in both *j*_Geo_ and product distribution, obscuring the true system behavior. This becomes especially problematic at higher currents since the misapplication of the applied potential scales with this parameter. By embedding operando EIS into a real-time Python feedback loop, our approach provides a practical solution: the experiment continuously corrects itself, maintains the intended potential, and stabilizes long-term testing under inherently dynamic conditions.

Therefore, the dynamic IR compensation approach enabled an accurate investigation of ECO_2_R stability at −1.025 V vs. RHE (Figure 3c). Over 2 h, the performance drop consisted of: rapid *j̅*_Geo_ decrease from −6.5 to −3.5 mA within the first 0.5 h, followed by a slower decline to −2.75 mA over the next 1.5 h; (ii) ethylene and methane FE ratios decreased in parallel, by ∼15% and ∼23% in the first 0.5 h and an additional ∼5% by the end of the test; (iii) CO and HER FE ratios decreased by ∼15% and ∼23% in the first 0.5 h with an additional ∼5% drop by the end of the experiment; and (iv) no drift in *R*_Ohm_ was observed. The ECO_2_R selectivity decrease at the cost of HER selectivity increase was consistent with previous reports.^35–57^ Similarly, the drop in *j*_Geo_ has also been observed in prior studies.^40,43,46,49,56,58^ However, the rate of performance drop from our study does not align with other studies. The general direction is the same among the investigations in the field, albeit none completely overlap due to the high complexity of the deactivation and different systems used. This underscores the importance of better understanding this behaviour,^42,59^ and provides further insight into the overpotential control presented in this study, elucidating a reliable degradation pathway.

On the other hand, the fluctuating behavior of *R*_Ohm_ without drift suggested that the electrolyte remains stable throughout the experiment, and that this effect didn’t contribute to the observed deactivation behavior. Moreover, the results from this study further support the findings that gas bubbles do not influence the electrochemical reactions occurring on the catalyst surface, with their sole effect being the altered *R*_Ohm_. This was the most evident in an experiment presented in Figure S12: at the interval between 2500 and 3500 s, the bubble distribution changed significantly, causing *R*_Ohm_ to shift from ∼35 to ∼22.5 Ohm. The GC analysis was performed during the static *R*_Ohm_ phases, where the IR compensation was correctly applied (Figure S12, fifth and sixth points), revealing that despite substantial changes in bubble distribution, ECO_2_R remained unaffected. Selectivities followed a smooth, continuous trend in this time period. If the bubbles’ distribution would affect the electrochemical reaction, some discrepancies would be observable in the *j̅*_Geo_ and selectivity evolutions. Therefore, the understanding of bubble-induced effects was further generalized to a completely different system.

While implementation of the approach on different reactions, systems, conditions, etc., would further prove the utility of the developed method and support the presented arguments, this exceeds the scope of this article. However, by providing instructions on the approach implementation (Section S4) and a proof of concept in a real dynamic example (Figure 3), this article aims to encourage other groups to implement it in their systems, tackling a variety of distinct problems. For example, a highly prominent information obtained from operando EIS is also the *R*_CT_ through which the catalyst-electrolyte interface could be monitored (Scheme 1). The advantage of our method over conventional operando EIS is the application of manual IR compensation even during the EIS measurements (Figure 3a). This is of utmost importance since *R*_CT_ is dependent on the applied potential.

The backbone of the Python code presented in the Supporting Information was originally written with almost no background in coding through the utilization of ChatGPT. As presented in the section on code implementation to other systems (Section S4), the use of ChatGPT (or other equivalent AI models) is encouraged to easily, quickly, and accurately modify the scripts. Especially since most of the potential users are probably not experts in coding, the utilization of AI models bridges this gap and allows everyone to modify the approach according to their own needs. Conventionally, a sophisticated and nice program should be provided; however, due to the high diversity of potential applications of the approach, we provide a simple script that could be modified without difficulty. If this approach is further employed, the generation of large datasets could facilitate the use of machine learning to accelerate both data interpretation and the identification of solutions, possibly achieving an even better control over the bubble dynamics in electrochemistry. Additionally, the self-correcting approach could be employed to change other electrochemical (or completely other) parameters based on results of other methods than the ones used in this study, further expanding the scope of this study beyond operando EIS.

## Conclusion

In summary, we developed a self-correcting operando EIS approach that enables dynamic IR compensation to mitigate bubble-induced distortions of the applied potential. By continuously extracting *R*_Ohm_ and adjusting the compensation in real time, our method stabilized the interfacial potential throughout extended ECO_2_R experiments. This ensured that performance metrics such as *j*_Geo_ and selectivity were measured at the true intended potential, free from artifacts caused by random bubble evolution. The resulting datasets allowed a clear evaluation of long-term ECO_2_R stability, where gradual declines in *j*_Geo_ and ethylene product selectivity could be reliably distinguished from bubble-driven fluctuations.

Beyond serving as a proof of concept, this study provides a practical framework for improving potential control in gas-evolving electrochemical systems. The Python-based feedback loop is intentionally simple and adaptable, lowering the barrier for implementation in other laboratories and systems. While alternative strategies exist to mitigate bubble effects, the integration of operando EIS into real-time experimental control represents an accessible and versatile route for stabilizing long-term measurements. More broadly, approaches like this, where feedback from diagnostic tools directly informs ongoing experiments, can improve the reliability of electrochemical stability studies and accelerate progress in electrocatalysis.

## Supplementary Material



## Notes

Detailed experimental section, additional electrochemical experiments, detailed approach, and code explanation with instructions for utilization (the Python scripts are available for download free of charge at https://github.com/ECatGroup/operando_impedance_spectroscopy) (PDF)
