# Supplementary material for: Operando Impedance Spectroscopy Informed Dynamic Internal Resistance Compensation Mitigates Bubble-Induced Distortions of the Applied Potential
Source: ACS Electrochem. 2025 Oct 14;1(12):2823–30. doi: 10.1021/acselectrochem.5c00368 (PMC12683638; doi:10.1021/acselectrochem.5c00368)
Supplement: Supplementary file 1 [file ec5c00368_si_001.pdf]

Supporting Information

Operando Impedance Spectroscopy Informed Dynamic IR Compensation Mitigates Bubble-Induced Distortions of the Applied Potential

Blaž Tomc[ab], Miha Hotko[ab], Aleš Marsel[ac], Nik Maselj[ac], Maja Svete[a], Luka Suhadolnik[a], Marjan Bele[a], Pedro Farinazzo Bergamo Dias Martins[a], Dušan Strmčnik[a]*, Miran Gaberščček[ac]*, Nejc Hodnik[abd]*

[a]Laboratory for Electrocatalysis, Department of Materials Chemistry, National Institute of Chemistry, Ljubljana 1000, Slovenia

[b]University of Nova Gorica, Nova Gorica 5000, Slovenia

[c]University of Ljubljana, Faculty of Chemistry and Chemical Technology, Ljubljana 1000, Slovenia

[d]Institute of Metals and Technology, Ljubljana 1000, Slovenia

email: *Nejc Hodnik: nejc.hodnik@ki.si, *Miran Gaberščček: miran.gaberscek@ki.si, *Dušan Strmčnik: dusan.strmcnik@ki.si, *Blaž Tomc: blaz.tomc@ki.si

Section S1: Experimental Section

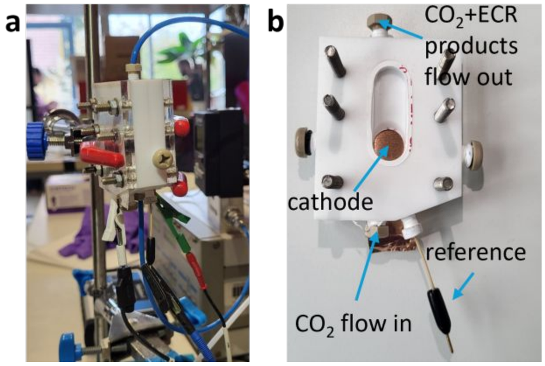

Figure S1: a) Assembled and b) half-cell utilized in this study.

Table S1: Measurement parameters for EIS experiments shown in Figures 2, 3b,c, and S10. Reported values include the reference electrode potential measured before and after the experiment vs. RHE in a 0.1 M $KHCO_3$, high and low frequency limits of the EIS scan, number of data points across the frequency range, and the applied AC amplitude (E_{AC}). These parameters were optimized to balance measurement time, signal quality, and spectral resolution depending on the experimental focus.

Experiment	Ref. bef. [V]	Ref. aft. [V]	High freq. [Hz]	Low freq. [Hz]	Points	E_{AC} [V]
Figure 2	-0.361	-0.364	25 000	250	21	0.010
Figure 3b	-0.361	-0.364	25 000	250	21	0.010
Figure 3c	-0.363	-0.362	25 000	250	21	0.020
Figure S11	-0.785	-0.789	30 000	300	24	0.006

These fluctuations arise from the random release of gas bubbles, which influence the product detection and are evident in Figure 1a. Because the GC analyzes only a 1 mL aliquot of the gas stream every 10 minutes, rather than the total volume of approximately 50 mL, the detected product ratios vary depending on the timing and extent of bubble release. If a major bubble release occurs coinciding with GC sampling, a higher product-to-CO_2 ratio is detected, leading to an artificially elevated FE

that can exceed 100% (Figure S2). By normalizing the total FE to 100%, the impact of these stochastic bubble removal events on the analysis was minimized.

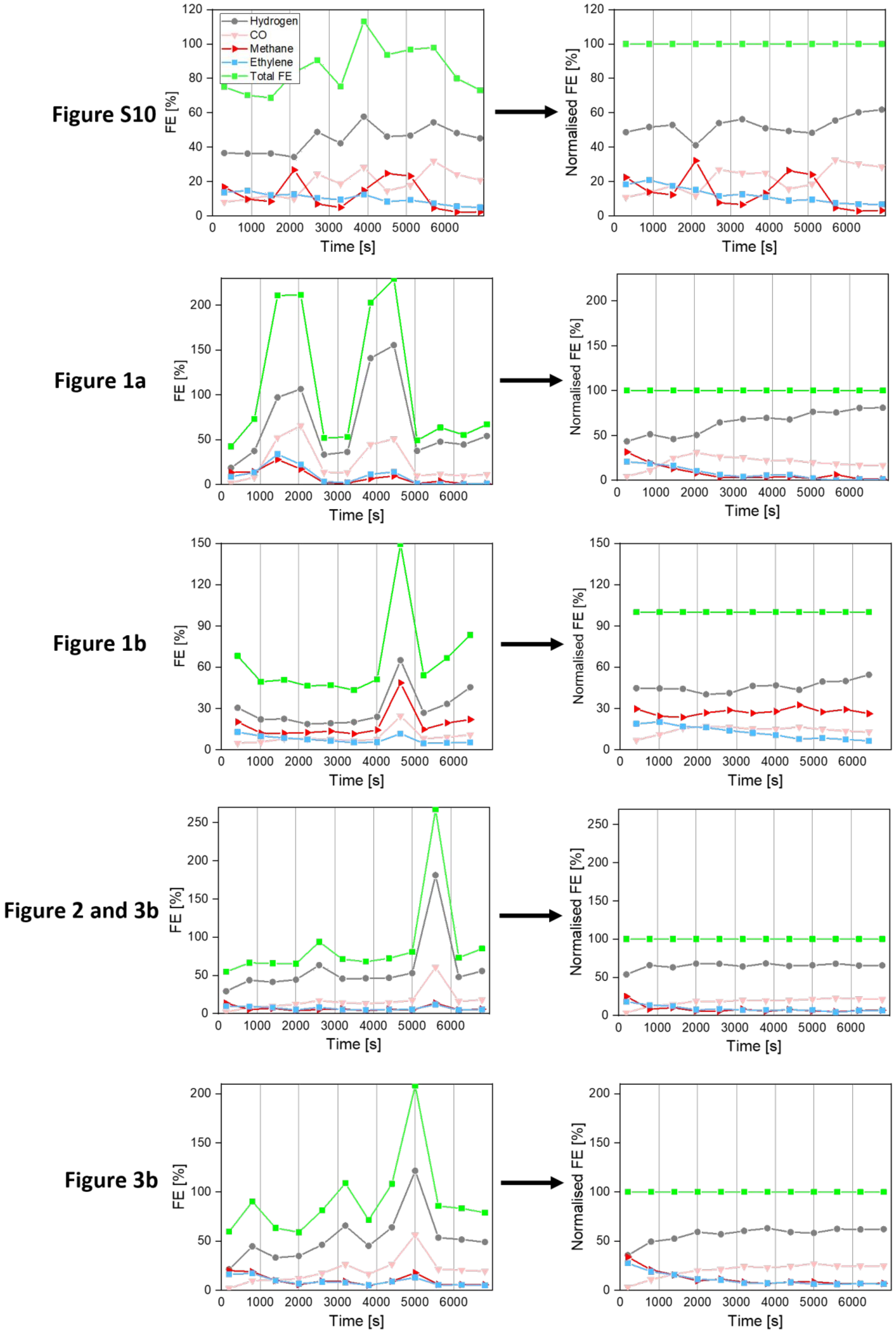

Figure S2: The measured FE and its normalization to 100% to limit the bubble-induced fluctuations.

Section S2: Detailed Approach Explanation

The operando EIS approach relied on a PSTrace script executing a loop of electrochemical techniques, while a Python program monitored and evaluated the results in real time. Based on the outputs, it dynamically adjusted the method parameters, ensuring consistent and accurate electrochemical conditions. The logic and execution of the measurement sequence are described below.

PSTrace loop of measurements

The approach consisted of 4 methods (Figure S3). To make sure the applied potential remains uninterrupted, the newest version of the PSTrace (>5.9.) should be used.

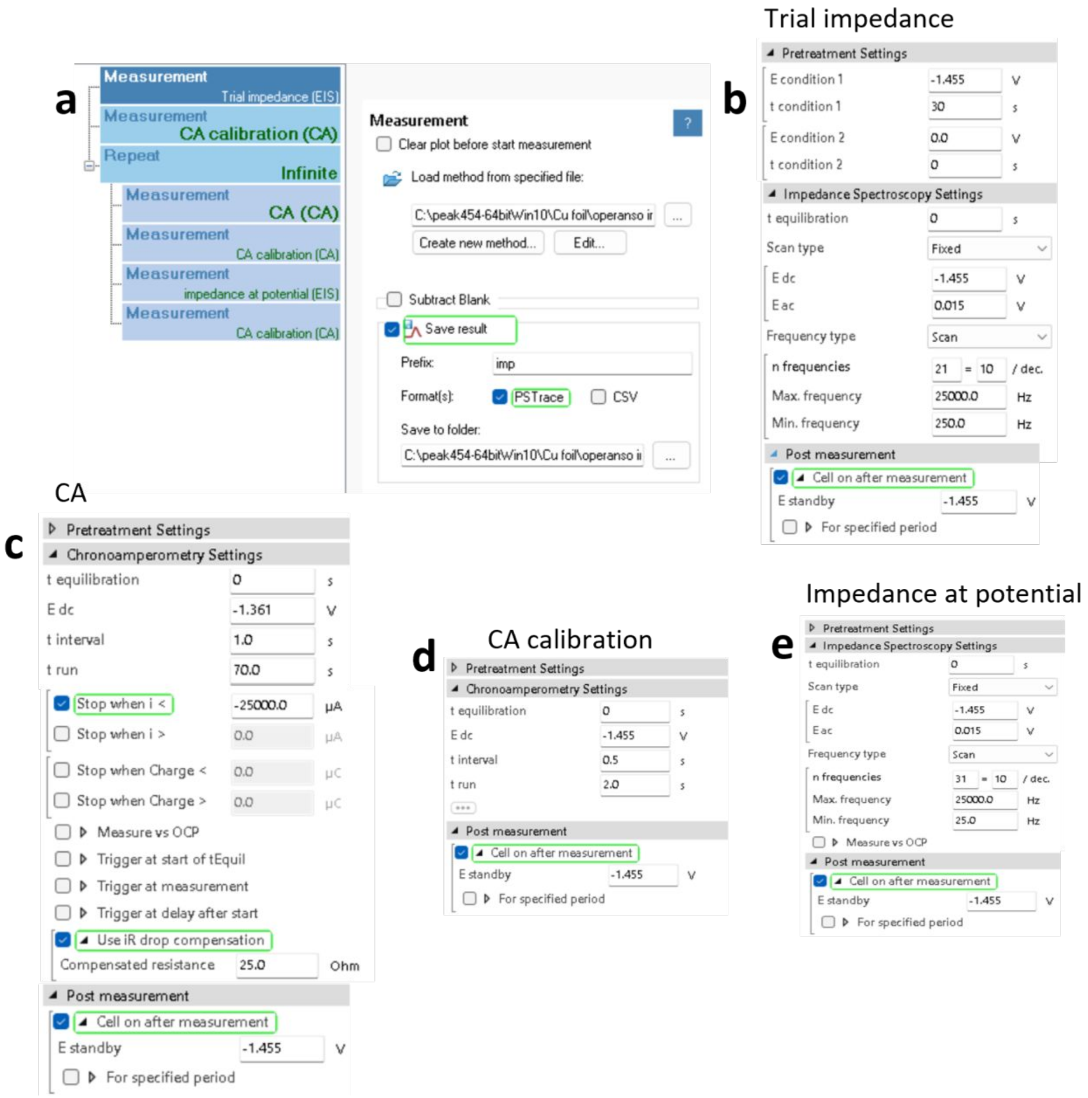

Figure S3: Graphical overview of the experimental method setup in PSTrace, demonstrating the automated loop of electrochemical techniques used for real-time self-correction. (a) Sequence of trial EIS, CA, and operando EIS steps, with CA and EIS methods embedded in an infinite repeat loop. On the right is presented the method configuration window, highlighting the saving of EIS results with the prefix “imp” for Python processing. (b–e) Individual method settings for pretreatment, IR compensation, potential control, and frequency range for both CA and EIS steps. These settings enable dynamic IR compensation and, when extended, potential adaptation based on real-time EIS feedback, ensuring consistent and accurate electrochemical conditions during extended CO_2 reduction experiments.

1) The loop begins with a trial EIS measurement, which includes a 30-second equilibration period at a potential that is not IR-compensated. However, if approximate values for R_{Ohm} and $\bar{I}$ are already known from earlier experiments, the Python script can adjust the applied potential accordingly before the measurement begins. This EIS is performed over a frequency range of approximately 30 kHz to 300 Hz using around 10 points per decade and a small AC amplitude (in the mV range).

This trial EIS primarily serves to determine the initial R_{Ohm}. Since R_{Ohm} is potential-independent, the lack of IR compensation at this point does not affect its accuracy. The EIS spectrum is saved with the prefix "imp" in a monitored directory (Figure S3a and Figure S8a,b), where it is processed by the Python script to extract R_{Ohm}.

The measurement parameters—such as frequency range, points per decade, and AC amplitude—can be freely adjusted by the user to suit their system. While frequency range and point density have no direct influence on the electrochemical reaction, they do affect the clarity of the Nyquist plot and the duration of the EIS measurement.[32] If the primary goal is to identify the breakpoint between R_{Ohm} and R_{CT} semicircles, the range can be narrowed and the number of points reduced to shorten acquisition time. However, it is essential that the chosen parameters still yield a distinct semicircle; the Python algorithm presented in this study requires at least six valid points around the breakpoint for accurate evaluation. In contrast, the AC amplitude must be selected carefully, especially for sensitive reactions like CO_2 reduction, where higher amplitudes can affect product selectivity. Therefore, it is generally kept as low as possible while maintaining an adequate signal-to-noise ratio.[32] Table 1 provides examples of suitable parameter sets used in this study (these are for the main EIS measurements).

2) Following the trial EIS, a 2-second chronoamperometry (CA) step is applied at the same potential. This brief CA is not saved but is used to allow the Python script enough time to evaluate the EIS results and update the method parameters for the upcoming steps.

3) Next, a main CA step lasting approximately 60 to 100 seconds (depends on the EIS) is performed at a potential corrected for the initial R_{Ohm} result of the trial EIS (100% IR compensation). This CA is used to evaluate current stability and performance and is saved with the prefix "CA" (Figure S8) in the specified directory. In addition, this CA has a smart handbrake if the system goes into oscillations due to large bubble removal, as observed in Figure S8. It does so by having ticked stop when $i < 25.000$ (Figure S3c). This is an optional step, but from our experiments, a very important one. The value can, of course, be adjusted freely. If triggered, it ends the measurement right away and goes to EIS, where the new R_{Ohm} is calculated. Otherwise, in normal cases, the duration of CA is as determined. The Python script processes the saved CA files, calculates the average current ($\bar{I}$), and prepares for the next steps.

4) Following the main CA, a second 2-second CA is applied at a potential corrected for the most recent R_{Ohm}. Like the initial 2-second CA, this step is not saved but serves to give the Python script time to process the main CA results, calculate the updated $\bar{I}$, and prepare parameters for the main EIS step. This additional pause ensures that all method files are updated before the EIS spectrum is recorded.

5) The main EIS step is then performed at a potential fully corrected for both R_{Ohm} and $\bar{I}$. Manual IR compensation is applied in PSTrace via the updated applied potential in the EIS method file. The EIS spectrum is acquired with a frequency range selected based on the measurement goal: (i) if only R_{Ohm} is needed, a higher low-frequency cutoff (e.g., 300 Hz) is used to reduce measurement time, and (ii) if R_{CT} is to be evaluated, the frequency range extends to lower limits (e.g., 15–35 Hz). See Table 1. The EIS data are saved with the prefix "imp" and immediately analyzed by the Python script to extract updated R_{Ohm} and R_{CT} values. Additionally, the frequency range, points per decade, and alternating potential can be adjusted by the user on-line by changing the method files manually, adding even more flexibility to the approach. So, if, e.g., the fluctuations are increasing with time of the measurement, causing Python to be unable to calculate the resistances, the AC voltage is increased by opening the method file in another PSTrace window, changing it, and overriding the previous method file.

6) To finalize the loop, a final 2-second CA is applied at the same IR-compensated potential as the main EIS step. As with previous short CAs, it is not saved but provides sufficient time for the Python script to process the latest EIS results and update parameters for the next loop iteration.

After the final 2-second CA, the loop resets and begins a new cycle (steps 3 to 6). All electrochemical methods (CA and EIS) in the PSTrace script are embedded within an infinite repeat loop (Figure S3a). The parameter updates at the usual flow are presented in Table S2 below.

Table S2: Overview of the electrochemical measurement sequence and the applied potentials at each step within the self-correcting operando EIS loop. The trial EIS establishes the initial R_{Ohm}, which is then used to calculate the corrected potential for subsequent CA and EIS steps. Short 2s CA measurements (labeled x) are used for synchronization and parameter adjustment but do not produce stored results. The main CA and EIS steps (labeled 1, 2, etc.) are conducted with updated R_{Ohm} and $\bar{I}$ values to maintain consistent electrochemical conditions. The table shows how applied potential (E), IR compensation, standby conditions, and resulting values (R_{Ohm}, R_{CT}, $\bar{I}$) evolve dynamically throughout the loop.

Method	E of conditioning	E of the methods	IR compensation	E standby	Result
Trial EIS (0)	$E = E_{CA} + R_{Ohm}^{Expected} \times I^{Expected}$	$E = E_{CA} + R_{Ohm}^{Expected} \times I^{Expected}$	/	$E = E_{CA} + R_{Ohm}^{Expected} \times I^{Expected}$	R_{Ohm}^{0}
2s CA calibration (x)	/	$E = E_{CA} + R_{Ohm}^{Expected} \times I^{Expected}$	/	$E = E_{CA} + R_{Ohm}^{Expected} \times I^{Expected}$	/
Main CA (1)	/	$E = E_{CA}$	R_{Ohm}^{0}	$E = E_{CA} + R_{Ohm}^{0} \times I^{Expected}$	I^{1}
2s CA calibration (x)	/	$E = E_{CA} + R_{Ohm}^{0} \times I^{Expected}$	/	$E = E_{CA} + R_{Ohm}^{0} \times I^{Expected}$	/
Main EIS (1)	/	$E = E_{CA} + R_{Ohm}^{0} \times I^{1}$	/	$E = E_{CA} + R_{Ohm}^{0} \times I^{1}$	R_{Ohm}^{1}, R_{CT}^{1}
2s CA calibration (x)	/	$E = E_{CA} + R_{Ohm}^{0} \times I^{1}$	/	$E = E_{CA} + R_{Ohm}^{0} \times I^{1}$	/
Main CA (2)	/	$E = E_{CA}$	R_{Ohm}^{1}	$E = E_{CA} + R_{Ohm}^{1} \times I^{1}$	I^{2}
2s CA calibration (x)	/	$E = E_{CA} + R_{Ohm}^{1} \times I^{1}$	/	$E = E_{CA} + R_{Ohm}^{1} \times I^{1}$	/
Main EIS (2)	/	$E = E_{CA} + R_{Ohm}^{1} \times I^{2}$	/	$E = E_{CA} + R_{Ohm}^{1} \times I^{2}$	R_{Ohm}^{2}, R_{CT}^{2}

This dynamic feedback loop ensures that the applied electrochemical conditions remain stable, even as the system evolves due to bubble formation, degradation, or other fluctuations. Because all method files are overwritten in each cycle, the approach is highly adaptable—offering a robust platform for long-term monitoring or stress testing under operando conditions (e.g., in the 10-hour pulsating experiment presented in Figure 4c,d, the PSTrace and Python worked seamlessly without breakdown).

In experiments designed to stabilize R_{CT} (Figure 3), the applied potential was dynamically modified based on the evolution of R_{CT} values during the loop. Specifically, the R_{CT} obtained from the second EIS measurement in the sequence was set as the reference. For each subsequent loop, the difference between the current R_{CT} and the reference R_{CT} was multiplied by a correction factor (typically 1/2500), and the resulting value was subtracted from the base potential.

For the pulsating experiment (Figure 4), the 2s CA after EIS (step 6) was substituted for the 2s CP at +1mA.

In addition to dynamic potential correction, the Python script was used to maintain strict loop duration control. Because EIS steps varied in length depending on the chosen frequency range, and when an oscillation occurs and the CA is shorter than usual, the script automatically adjusted the duration of the following CA step to ensure each full loop lasted exactly 120 seconds. This precise timing ensured that gas chromatographic (GC) sampling occurred consistently during the main CA step, maintaining alignment between electrochemical data and gas-phase product analysis.

While the parameter choices (e.g., frequency limits, timing, feedback factors) were optimized for the CO_2 reduction system studied here, they can be adapted for other electrochemical setups with minimal modification.

Python code

The Python code is structured with three separate threads, including a main thread, and several supplementary functions that enable the program to operate effectively. In our experiments, we have run it in Python's default Integrated Development and Learning Environment (IDLE). The Python code consists of several features divided into subsections:

1. Parameter updater function

This function modifies specific parameters in a settings file used for electrochemical experiments (the ones that PSTrace uptakes for a measurement). The method files are encoded in UTF-16 and contain distinct parameters such as IR_DROP_COMP_RES, T_RUN, E, E_BEGIN, E_STBY, and CURRENT, which are important for controlling experimental conditions (Figure S4a).

The function is called in the main thread with different arguments in the brackets, such as the file path and parameters that need to be changed. The function opens the file at the specified file path, searches for the parameters that need changes, and updates them with new values if provided. It ensures that numerical values are formatted in scientific notation (e.g., 1.23E+02).

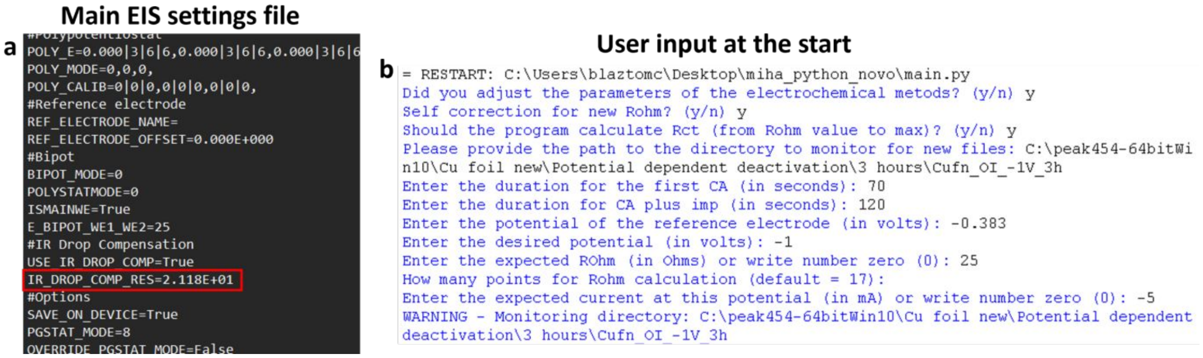

Figure S4: a) Screenshot of the part of the main EIS settings file where the IR drop compensation parameter is set. b) Screenshot of the start of the script in IDLE. In the blue text, the Python inputs are written, while the black text represents the user input.

2. Pre-measurement parameter change

This part of the main thread sets up the experiment by gathering user input and updating key parameters in method files used by the electrochemical instrument. It first asks the user to confirm whether they have adjusted the experimental settings (especially for EIS) and then prompts them to enter the directory where data files will be monitored. The user also provides important experimental values, such as reference electrode potential, desired working potential, times of different methods, expected resistance, and current (Figure S4b). Using these inputs, the script calculates the correct potential (as presented in step 1) and, if necessary, adjusts it based on the expected resistance and current. Finally, the script updates the .psmethod files with the parameter updater function, ensuring that the correct potential and duration values are set before the experiment begins. This automation helps streamline the setup process, reducing manual adjustments and minimizing errors.

3. Directory monitoring

After the parameters are updated, the observer thread continuously monitors a specified directory for new files, particularly those related to CA ("CA" prefix) and impedance ("imp" prefix) measurements. The observer thread and functionality are provided by the Python module "watchdog". When a new file appears, the program waits for it to stabilize (ensuring it's fully written) and then processes it. The program has two different processing loops for further calculation, depending on whether the file has the prefix "CA" or "imp".

4. Time function

As mentioned, the durations of EIS techniques vary. To ensure that GC analysis is performed precisely at the same time as CA and to track when the results occur, a global timer was implemented in the main thread (state). It starts when the first "imp" file is saved in the directory. If the interval deviates from the user-defined time set at the beginning, the code adjusts the duration of the next main CA accordingly. This process is repeated for all subsequent files.

5. Impedance data processing

The impedance data processing is initiated by the process thread to analyze EIS data with the "imp" prefix. While this pipeline is optimized for the current system, adapting it to other setups may require significant modification—especially due to differences in file structure depending on the potentiostat and its software version.

The IR_calculation routine uses the extract_data_imp_new function to analyze impedance spectra. The raw data is extracted from UTF-16 encoded JSON files using Python's json module. Real (ZRe) and imaginary (ZIm) impedance values are retrieved

and cleaned, including removal of negative values. Specific filtering logic is applied to retain only meaningful points: this involves analyzing local trends in ZRe to identify and exclude inconsistent points.

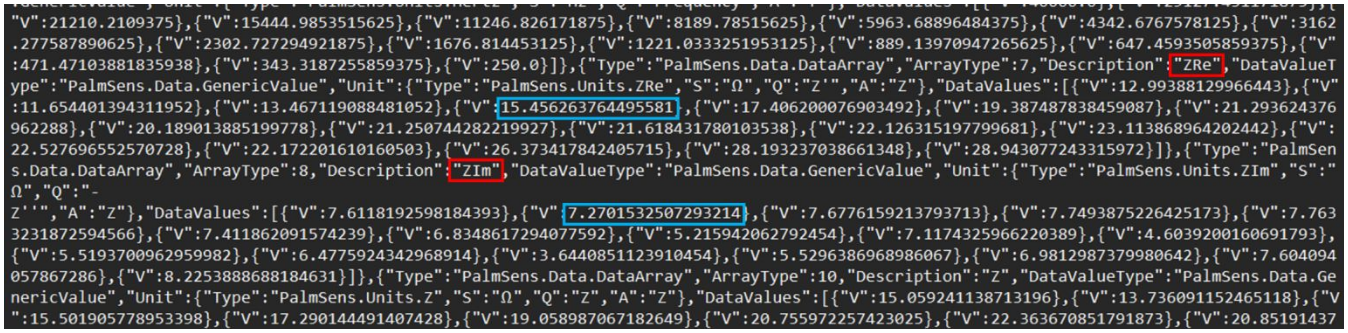

"V":21210.2109375},{"V":15444.9853515625},{"V":11246.826171875},{"V":8189.78515625},{"V":5963.68896484375},{"V":4342.6767578125},{"V":3162
.277587890625},{"V":2302.727294921875},{"V":1676.814453125},{"V":1221.0333251953125},{"V":889.13970947265625},{"V":647.4593505859375},{"V"
:471.47103881835938},{"V":343.3187255859375},{"V":250.0}]},{"Type":"PalmSens.Data.DataArray","ArrayType":7,"Description":"ZRe","DataValueT
ype":"PalmSens.Data.GenericValue","Unit":{"Type":"PalmSens.Units.ZRe","S":"Ω","Q":"Z'","A":"Z"},"DataValues":[{"V":12.9938812966443},{"V"
:11.654401394311952},{"V":13.467119088481052},{"V":15.456263764495581},{"V":17.406200076903492},{"V":19.387487838459087},{"V":21.293624376
962288},{"V":20.189013885199778},{"V":21.250744282219927},{"V":21.618431780103538},{"V":22.12631519779681},{"V":23.11386896420244 2},{"V":
22.527696552570728},{"V":22.172201610160503},{"V":26.373417842405715},{"V":28.193237038661348},{"V":28.943077243315972}]},{"Type":"PalmSen
s.Data.DataArray","ArrayType":8,"Description":"ZIm","DataValueType":"PalmSens.Data.GenericValue","Unit":{"Type":"PalmSens.Units.ZIm","S":"
Ω","Q":"-
Z''","A":"Z"},"DataValues":[{"V":7.6118192598184393},{"V":7.2701532507293214},{"V":7.6776159213793713},{"V":7.7493875226425173},{"V":7.763
3231872594566},{"V":7.411862091574239},{"V":6.8348617294077592},{"V":5.215942062792454},{"V":7.1174325966220389},{"V":4.6039200160691793},
{"V":5.5193700962959982},{"V":6.4775924342968914},{"V":3.6440851123910454},{"V":5.5296386968986067},{"V":6.9812987379980642},{"V":7.604094
057867286},{"V":8.2253888688184631}]},{"Type":"PalmSens.Data.DataArray","ArrayType":10,"Description":"Z","DataValueType":"PalmSens.Data.Ge
nericValue","Unit":{"Type":"PalmSens.Units.Z","S":"Ω","Q":"Z","A":"Z"},"DataValues":[{"V":15.059241138713196},{"V":13.736091152465118},{"V
":15.501905778953398},{"V":17.290144491407428},{"V":19.058987067182649},{"V":20.755972257423025},{"V":22.363670851791873},{"V":20.85191437

Figure S5: Screenshot of the part of the EIS result file where the Nyquist plot data are. The red rectangle represents the array identifier, while in blue, one of the data is highlighted.

A backward filter ensures continuity in ZRe across the spectrum, followed by smoothing via moving-point averaging over several iterations to suppress artifacts, such as those caused by gas bubbles. A polynomial fit to the cleaned data is then used to identify the minimum of ZIm—a critical point that corresponds to the system's characteristic frequency.

If this minimum is not well-defined or lacks consistent neighboring data, the function applies higher-order polynomial fits (2nd to 4th degree) and derivative-based root analysis to more reliably locate true impedance features. Based on the fit, it returns the refined ZRe value at the minimum of ZIm, which is used to calculate the R_{Ohm}. If no sufficient data or a minimum can be identified, the function exits and retains the last valid R_{Ohm} value from previous measurements.

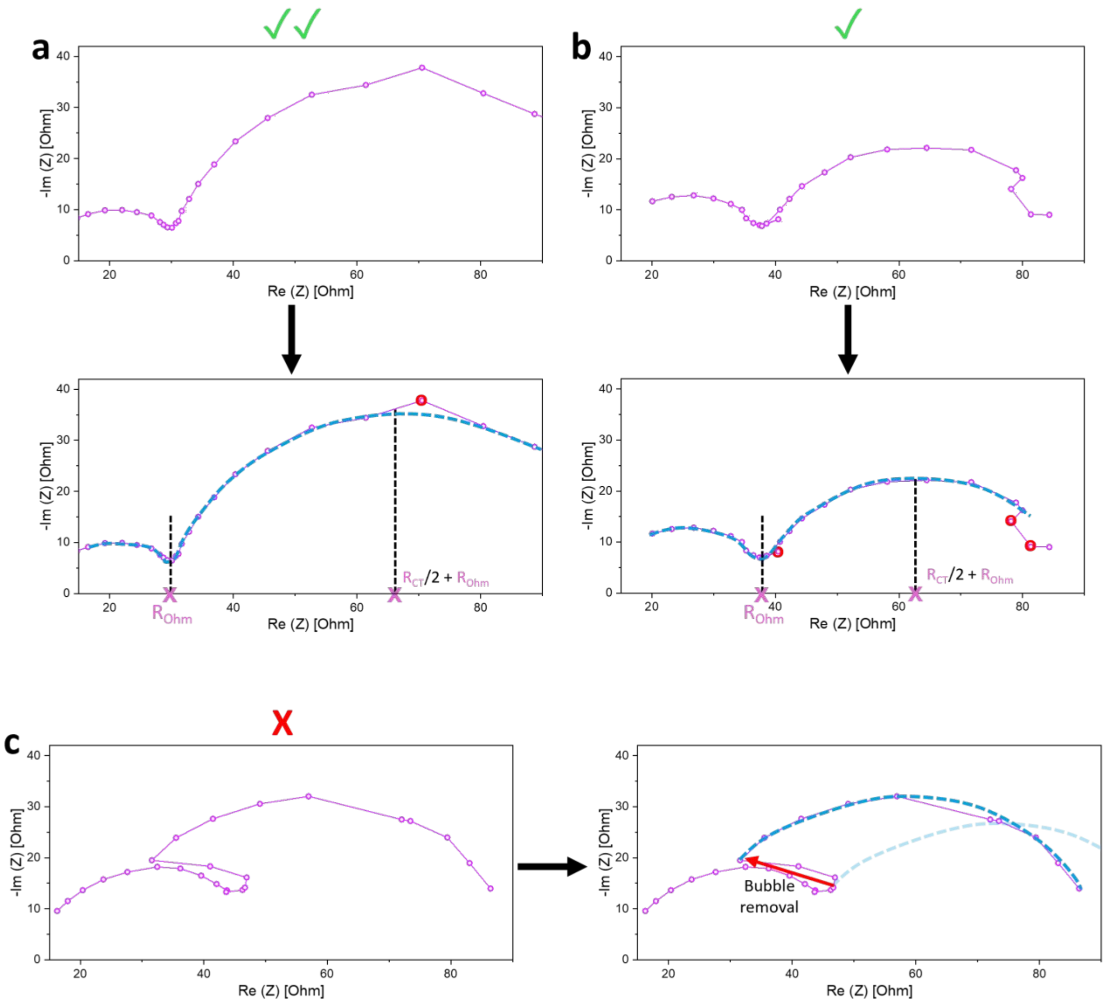

Figure S6: Illustration of how the Python script processes impedance spectra to extract R_{Ohm} and R_{CT} values. a) well-formed spectra that enable reliable fitting and point detection. b) Example where the script correctly identifies R_{Ohm} and R_{CT} despite

mild distortions. The dashed blue curves represent fitted semicircles, while red points mark values that are outliers. c) A failed EIS experiment where there was a significant bubble change during the measurement. This result is excluded from analysis. The algorithm requires at least six distinct and correctly spaced data points near the breakpoint to extract R_{Ohm} and ensure robustness across varying spectral quality.

6. Chronoamperometry data treatment function

The extract_data_ca function is called up by the processing thread to extract and process the main CA data from a file, calculating the average current and its standard deviation. It starts by reading the file with UTF-16 encoded JSON. Once the JSON data is extracted, the function navigates through its structure to locate current readings under Measurements -> DataSet -> Values. It searches for an entry where the description is "current" and the data type matches PalmSens' current readings (Figure S7). If no such data is found, the function exits with an appropriate message.

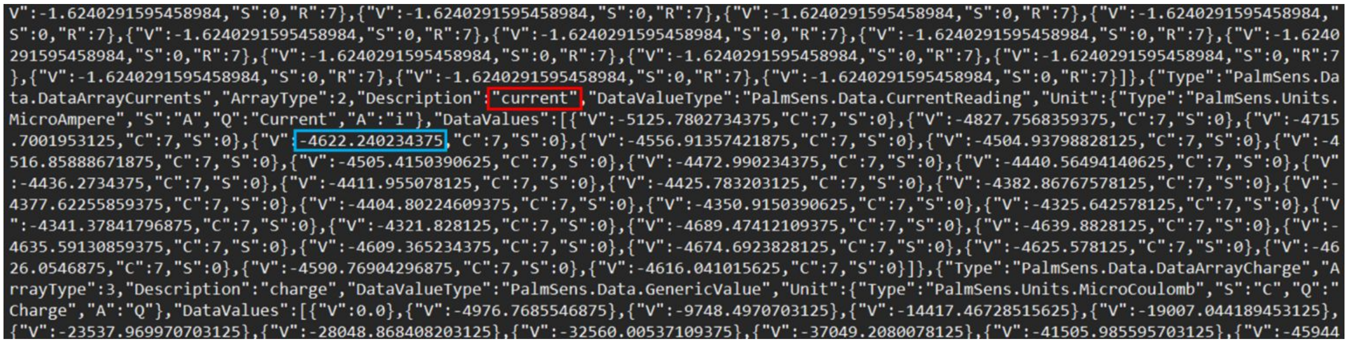

V":-1.6240291595458984,"S":0,"R":7},{"V":-1.6240291595458984,"S":0,"R":7},{"V":-1.6240291595458984,"S":0,"R":7},{"V":-1.6240291595458984,"
S":0,"R":7},{"V":-1.6240291595458984,"S":0,"R":7},{"V":-1.6240291595458984,"S":0,"R":7},{"V":-1.6240291595458984,"S":0,"R":7},{"V":-1.6240
291595458984,"S":0,"R":7},{"V":-1.6240291595458984,"S":0,"R":7},{"V":-1.6240291595458984,"S":0,"R":7},{"V":-1.6240291595458984,"S":0,"R":7
},{"V":-1.6240291595458984,"S":0,"R":7},{"V":-1.6240291595458984,"S":0,"R":7},{"V":-1.6240291595458984,"S":0,"R":7}]},{"Type":"PalmSens.Da
ta.DataArrayCurrents","ArrayType":2,"Description":"current","DataValueType":"PalmSens.Data.CurrentReading","Unit":{"Type":"PalmSens.Units.
MicroAmpere","S":"A","Q":"Current","A":"i"},"DataValues":[{"V":-5125.7802734375,"C":7,"S":0},{"V":-4827.7568359375,"C":7,"S":0},{"V":-4715
.7001953125,"C":7,"S":0},{"V":-4622.240234375,"C":7,"S":0},{"V":-4556.91357421875,"C":7,"S":0},{"V":-4504.93798828125,"C":7,"S":0},{"V":-4
516.85888671875,"C":7,"S":0},{"V":-4505.4150390625,"C":7,"S":0},{"V":-4472.990234375,"C":7,"S":0},{"V":-4440.56494140625,"C":7,"S":0},{"V"
:-4436.2734375,"C":7,"S":0},{"V":-4411.955078125,"C":7,"S":0},{"V":-4425.783203125,"C":7,"S":0},{"V":-4382.86767578125,"C":7,"S":0},{"V":-
4377.62255859375,"C":7,"S":0},{"V":-4404.80224609375,"C":7,"S":0},{"V":-4350.9150390625,"C":7,"S":0},{"V":-4325.642578125,"C":7,"S":0},{"V
":-4341.37841796875,"C":7,"S":0},{"V":-4321.828125,"C":7,"S":0},{"V":-4689.47412109375,"C":7,"S":0},{"V":-4639.8828125,"C":7,"S":0},{"V":-
4635.59130859375,"C":7,"S":0},{"V":-4609.365234375,"C":7,"S":0},{"V":-4674.6923828125,"C":7,"S":0},{"V":-4625.578125,"C":7,"S":0},{"V":-46
26.0546875,"C":7,"S":0},{"V":-4590.76904296875,"C":7,"S":0},{"V":-4616.041015625,"C":7,"S":0}]},{"Type":"PalmSens.Data.DataArrayCharge","A
rrayType":3,"Description":"charge","DataValueType":"PalmSens.Data.GenericValue","Unit":{"Type":"PalmSens.Units.MicroCoulomb","S":"C","Q":"
Charge","A":"Q"},"DataValues":[{"V":0.0},{"V":-4976.7685546875},{"V":-9748.4970703125},{"V":-14417.46728515625},{"V":-19007.044189453125},
{"V":-23537.969970703125},{"V":-28048.868408203125},{"V":-32560.00537109375},{"V":-37049.2080078125},{"V":-41505.985595703125},{"V":-45944

Figure S7: Screenshot of the part of the CA result file where the current data are. The red rectangle represents the array identifier, while in blue, one of the current data points is highlighted.

If the current data is available, it then computes the average current and standard deviation for the measured values. The standard deviation is calculated using the Python module “numpy” and its function “std”. The additional statement was added to handle system oscillations: if the last value is lower than -25 mA (or adjusted accordingly), it excludes it, and if the length of the data file is less than 5 (the bubble change occurred at the beginning), it takes the previous current value. If any errors occur during JSON parsing or data extraction, the function handles them gracefully by logging an error message and returning None.

7. Live corrections

When a new CA file is detected, the process thread calls the extract_data_ca function to determine the I_{Geo} and standard deviation. The results are always converted to milliamperes and stored in shared data structures. Using this I_{Geo} and last R_{Ohm}, the thread adjusts the applied potential of EIS and 2s CA (E_begin) for IR drop compensation, ensuring consistent electrochemical conditions.

For EIS files, the main thread processes the detected file by IR_calculation, which gives back the R_{Ohm} resistance. This value is directly used to change the IR drop compensation in the CA measurement (Figure S8) and to change the applied potentials of subsequent 2s CA and EIS measurements. If a valid IR measurement is not found, the function retains the most recent valid result, ensuring stable and consistent parameter adjustments.

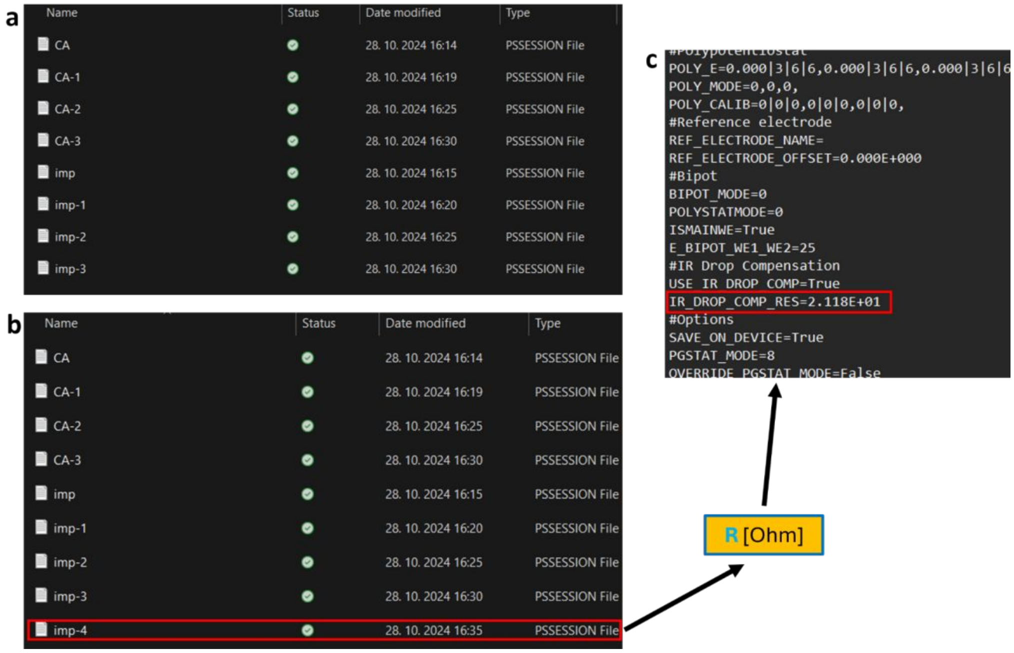

Figure S8: a) Screenshot of the directory where PSTrace saves the results with “CA” and “imp” prefixes. After a new file is saved in the directory, as presented with a red rectangle in b), Python calculates and evaluates the result and changes the settings file of the method as presented with a red rectangle in c).

By running continuously in a background thread, the observer thread ensures that live data is used to refine experimental conditions, maintaining accuracy and precision without requiring manual intervention.

8. Live graph

To have an overview of the data, live graphing is implemented in the script, based on different datasets.

9. Termination

To ensure simplicity, the code is simply terminated in IDLE by “Ctrl” + “c”.

10. Data saving

The plots and data are saved automatically when new files are processed, in the same folder as the program is observing during the measurement.

Section S2: Supplementary Electrochemical Experiments

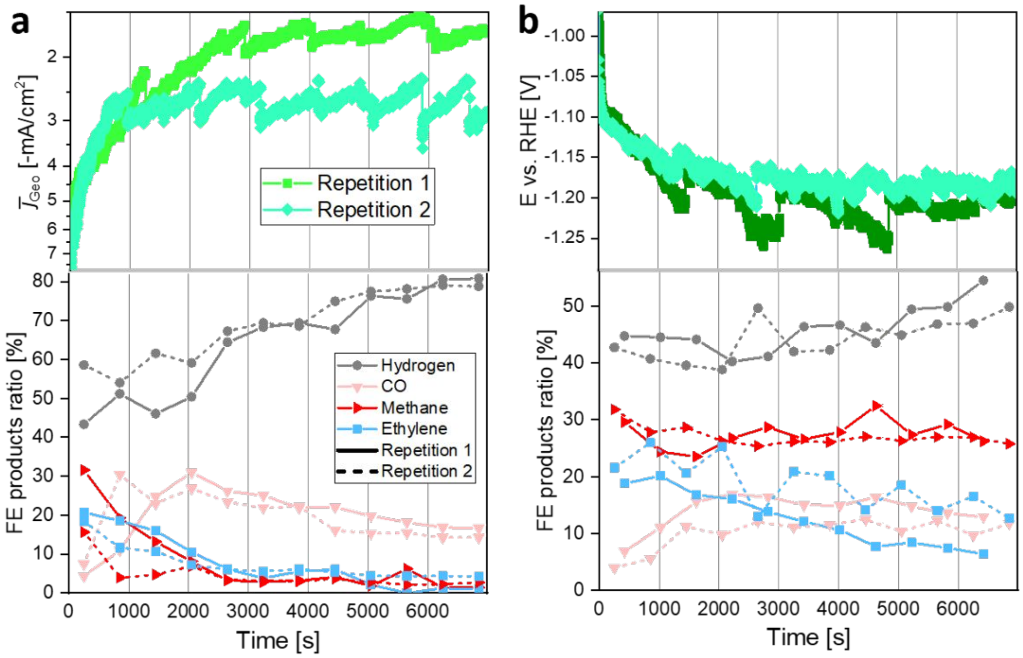

Figure S9: Repetitions of Figure 1a and b experiments under identical conditions.

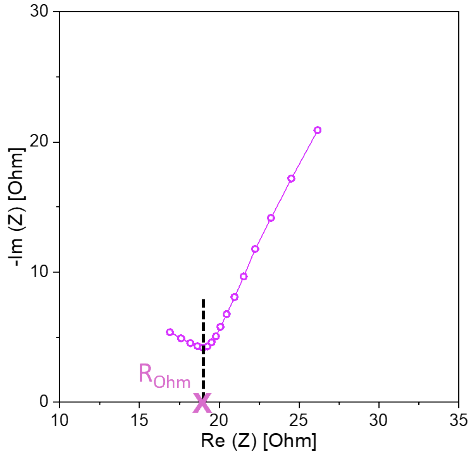

Figure S10: EIS measured without observable bubbles on the cathode-reference path.

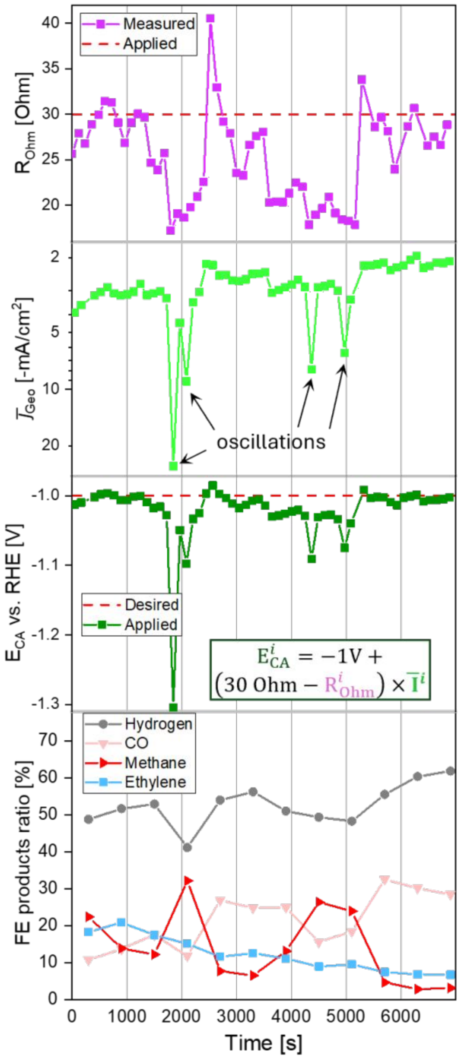

Figure S11: Operando EIS with static 30 Ohm IR compensation.

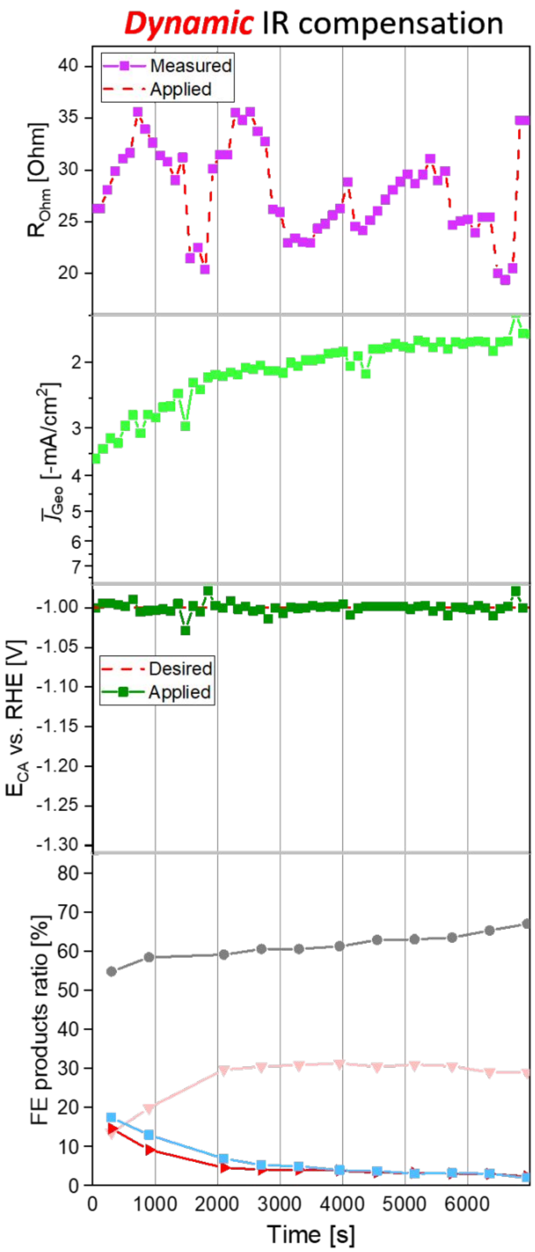

Figure S12: ECO_2R stability test at -1 V vs. RHE with dynamic IR compensation.

Section S4: Code Utilization and Modifications

This Python program is designed for use with PalmSens4 and the PSTrace software (>5.9.), as it is specifically written to read and modify .psmethod and .pssession files. If a different potentiostat or software is being used, the code must be adapted accordingly. Users can provide their method and result files, along with the provided Python scripts, to AI models to assist in reprogramming the scripts for compatibility with their specific system, as presented later in this section.

Protocol for implementation

To implement dynamic IR compensation within a similar setup to ours, begin by downloading the provided folder. If Python is not already installed, ensure that it is properly set up. The script “main.py” should then be opened in an editor that allows the execution of Python scripts, such as IDLE.

Before running the script, the required measurement methods must be created and configured in PSTrace (Figure S3b-e). The four necessary methods should be saved in the same folder with distinct names (Figure S13). If the user has changed the names from the default, which are shown in Figure S13, the names also need to be changed in the script “state.py”. The first EIS measurement requires specific settings: pretreatment should be enabled with a duration of 30 s (or adjusted as needed), but the potentials for pretreatment and DC voltage could remain unset, as Python will adjust these automatically before the measurement starts. The key EIS parameters that must be set manually are the frequency range, points per decade, and alternating voltage. A typical range of 30 kHz to 300 Hz, with 10 points per decade, was sufficient for identifying the transition between two semicircles in the case example. The alternating voltage parameter depends on the system and current levels, so a few test cycles should be performed to determine the optimal setting. In our system, alternating potentials between 3 and 20 mV were used.

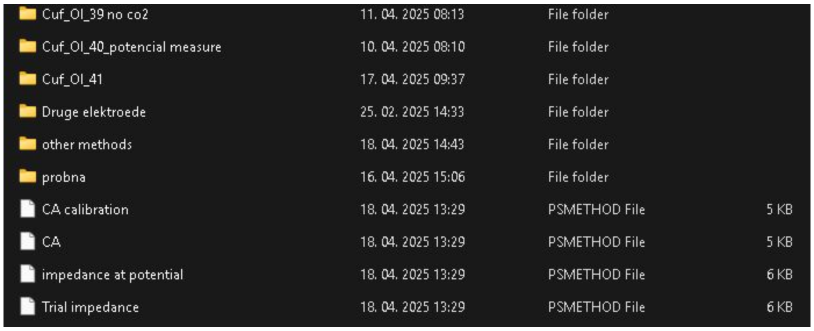

Figure S13: Directory with the saved .PSMETHOD files.

In the main CA method, IR compensation must be enabled, while its value, along with the measurement time and potential, will be set dynamically by the script. A second CA calibration method should also be prepared for use in a loop, but with the time parameter adjusted to 2 seconds (or adjusted accordingly). The loop EIS measurement should be configured similarly to the first EIS measurement, with the exception that pretreatment must be disabled. These file names must be formatted correctly and listed in the following order in the script “state.py”: main CA, 2-second CA, main EIS, trial EIS.

After configuring the methods, open the PSTrace software and create the measurement sequence. Six measurements should be added in sequence, selecting the corresponding methods in the same order as listed in Section 4 (trial EIS, 2s CA, main CA, 2s CA, main EIS, 2s CA). A repeat function should then be inserted, enclosing the last four methods, to define the looping sequence (Figure S3a). This loop can be set to a finite number of repetitions or to run indefinitely. It is important to enable the saving of EIS and main CA results and specify the directory where the results will be stored. The script requires EIS results to be saved with the prefix "imp" and CA results with the prefix "CA" (Figure S3a). If different names are preferred, these must be changed within the script “workdelegator.py” in the function process_file.

With everything set up, the main thread can be run. The script will prompt the user to enter the directory where measurement files will be saved, and Python will monitor this directory for new files. It will also ask for the measurement parameters (Figure S4b). Once these values are provided, the script will adjust the parameters of the methods inputted by the user, and then continuously monitor the folder and wait for the first EIS result file to appear (Figure S8).

Once PSTrace is running, Python will automatically update the measurement parameters (time, applied potential, IR compensation), handling all necessary adjustments throughout the experiment (Table S2). The script will run until manually terminated with a KeyboardInterrupt error by pressing Ctrl + C.

In addition to Python modifying the setting of the electrochemical measurements, parameters such as frequency range, points per decade, and alternating voltage can be adjusted on-line by the user. By opening the method in a second PSTrace, changing some of these parameters, and saving it with the same name (replacing the existing one) user can adjust the EIS measurements during the approach if some changes occur. For example, if the current increases, the signal-to-noise will be lower with time, producing unreadable spectra. By increasing the alternating voltage during the measurement user can

increase the signal-to-noise ratio to ensure the spectra is readable. When adjusting the range, care should be taken for the EIS time to not exceed the total loop time.

Limitations and needed modifications for further implementation

There are a few limitations that need to be satisfied, or the Python script needs to be modified (with programming experience, the assistance of AI models, or both)

1) The time of each loop (CA, 2s CA, EIS, 2s CA) could be adjusted by the will of the user. However, the time of the measurements needs to be considered. EIS measurement from 30k to 300 Hz at PalmSens4 takes around 30 s to execute. Therefore, to achieve a reliable CA measurement, it is recommended that the whole loop last at least 60 s. Similarly, if one wishes to observe the spectral range up to 0.1 Hz, the time of the loop should be adjusted accordingly. If the time of EIS exceeds the time of the loop, the code will crash. This is adjusted when the Python script is run and the program asks for the time of the loop.

2) The extract_data_imp_new function for finding the R_{Ohm} value is prepared to analyze spectra similar to the ones presented in this study (Figure S6). It needs at least 6 points that follow some order around the breaking point between the two semicircles. Since the most reliable results were achieved in the 30k to 300 Hz range with ~10 points per decade, the function was written in such a manner. The function has a built-in statement to take only the first 17 points in the EIS spectra, because if one wishes to observe the R_{CT}, the script might get confused by the breaking point of the R_{CT} and apply the wrong IR drop compensation. If one wishes to have more/fewer points, different ranges, etc., this can be modified when the script asks the user at the beginning. In theory, the script should be robust enough to handle any size of the R_{Ohm}, but there is an additional statement for our experiments that if the R_{Ohm} is not in the 0 to 50 Ohm range, it takes the last valid value to eliminate some potential big miscalculations (this can, of course, be adjusted). However, if one has more complex spectra, the spectra analysis code needs to be completely modified to satisfy the needs. Especially, if the reference electrode artifact is not observed, the extract_data_imp_new function should be modified with the logic that will extract the correct R_{Ohm} in the spectra.

3) This code was written to apply 100% IR drop compensation, which is unconventional for most electrochemical systems. If one wishes a conventional 85%, 90%, etc., the factor can be simply added in the script "workdelegator.py" before it returns the IR drop compensation changes.

The presented approach works well in our system. If you use the same equipment as ours and follow the above-mentioned steps, it should result in a successful implementation. However, for the implementation with different equipment, adjustments in the scripts would have to be made. This would mean that the user should have at least basic programming knowledge to adjust the code. However, by utilizing AI models, the need for extensive programming knowledge can be significantly reduced. First, the approach should be well understood by reading this document. Then it's advised to provide the Python scripts to the AI model and ask it to explain its workings. After that, one should ask AI what changes would be needed to implement the code in your specific system and follow its guidance.

In our case, the method files were inspected first by us and then provided to the AI model to create appropriate code to read the file and change the desired parameters. This was a trial-and-error approach by changing the Python script and running it on the "blank" experiment files. Then, in the same manner, the result files were presented to the AI model, and the intention was stated. After many unsuccessful attempts, the code was slowly built to its current status, where it can now seamlessly work in our system (Figure 4). It has proven very useful to provide any errors noted by Python directly to the AI model to quickly find a solution.

On the other hand, if the user wants to implement the approach on different electrochemical techniques, it could be done similarly to the implementation in other systems. By providing the method and result files and stating the desired utilization of the codes to the AI model, the script should be built by trial and error until reaching the desired functions.
